# Synergistic anti-inflammatory effects of the aqueous extract from *Desmodium heterophyllum* and photobiomodulation in a carrageenan-induced paw edema model

**DOI:** 10.1007/s10103-026-04822-7

**Published:** 2026-02-21

**Authors:** Tássio Malber Oliveira, Diego Mendes Xavier, Gracimério José Guarneire, Nerilson Marques Lima, Roberta Maria Abrão, Luiz Gustavo Vilela Filho, Brendha Sena Costa, Sandra Bertelli Ribeiro Castro, Caio César de Souza Alves, Alessandra de Paula Carli, Murilo Xavier Oliveira

**Affiliations:** Universidade Federal dos Vales do Jequitinhonha e Mucuri, Diamantina, Brazil

**Keywords:** Desmodium heterophyllum, Photobiomodulation, Cytokines, Synergistic effect, Low-level laser therapy

## Abstract

Inflammation is a complex biological response that, when uncontrolled, contributes to the development and worsening of several pathological conditions. Identifying safe and effective anti-inflammatory strategies remains an important therapeutic challenge. In this context, medicinal plants and photobiomodulation therapy (PBM) have emerged as promising approaches due to their immunomodulatory potential. This study aimed to investigate the anti-inflammatory properties of the aqueous extract from *Desmodium heterophyllum* (AEDh), alone and in combination with PBM, using a carrageenan-induced paw edema model. AEDh was obtained, chemically characterized through high-resolution mass spectrometry, and evaluated for cytotoxicity and nitric oxide (NO) production in J774A.1 and RAW 264.7 macrophages. In vivo anti-inflammatory activity was assessed in BALB/c mice subjected to carrageenan-induced paw edema and treated with AEDh (50–250 mg/kg), PBM (780 nm, 70 mW, 35 J/cm²), or their combination. Paw thickness was measured for up to 4 h post-induction, and cytokine levels (IL-1β, IL-6, TNF, IFN-γ) were quantified by enzyme-linked immunosorbent assay (ELISA). AEDh showed no cytotoxicity at the concentrations tested and significantly reduced NO production in vitro. In vivo, both AEDh and PBM reduced paw edema, with the combined treatment—especially AEDh at 250 mg/kg—producing a more pronounced effect, comparable to dexamethasone. The combination also markedly reduced pro-inflammatory cytokines, particularly IL-1β and IL-6, supporting a synergistic action. AEDh demonstrated relevant anti-inflammatory and immunomodulatory activity, which was further enhanced when combined with PBM. These findings highlight the therapeutic potential of integrating herbal medicine and photobiomodulation as a safe and effective strategy for managing inflammatory conditions. Keywords: Desmodium heterophyllum; Photobiomodulation; Cytokines; Synergistic effect; Low-level laser therapy.

## Highlights

- First study to demonstrate the synergistic anti-inflammatory effects of *Desmodium heterophyllum* extract combined with photobiomodulation;
- Significant reduction in edema and pro-inflammatory cytokines comparable to dexamethasone;
- Extract exhibited excellent biocompatibility, with no cytotoxicity to macrophage cell lines;
- Findings support the development of safe and effective integrative therapeutic strategies for inflammatory conditions.

## Introduction

Inflammation is a complex biological process that is essential for protecting the body, as it eliminates pathogens, removes damaged tissue, and helps re-establish homeostasis [1, 2]. Despite being a vital protective mechanism in the acute phase, chronic inflammation is associated with the development of several diseases with high morbidity and mortality, such as rheumatoid arthritis, cardiovascular disease, cancer, type 2 diabetes, and neurodegenerative disorders [3, 4].

Conventional therapeutic strategies for the management of inflammation include steroidal anti-inflammatory drugs (SAIDs) and non-steroidal anti-inflammatory drugs (NSAIDs). SAIDs inhibit the expression of phospholipase A2 and cyclooxygenase (COX)−2), while NSAIDs block the synthesis of prostaglandins and thromboxanes via the inhibition of cyclooxygenases (COX-1 and COX-2) [5, 6]. Although effective, these drugs have significant side effects, such as gastritis, ulcers, cardiovascular complications, kidney failure, and immunosuppression, which limit prolonged use and have motivated the search for safer therapeutic alternatives [7].

Herbal medicines have gained prominence as natural, safe, less expensive therapeutic options. It is estimated that more than 80% of the world’s population use traditional medicine as their main source of treatment, revealing the importance of herbal medicine for public health [8]. Among the medicinal plants of interest, *Desmodium heterophyllum* is reported to have anti-inflammatory, antioxidant, antifungal, antimicrobial, and neuroprotective effects, as indicated in modern pharmacological studies [9–11].

Photobiomodulation (PBM), also known as low-level laser therapy, has been increasingly investigated as a non-invasive and safe approach for modulating inflammation and promoting tissue repair. Its mechanism of action is primarily related to the absorption of photons by cytochrome c oxidase in the mitochondrial respiratory chain, leading to increased adenosine triphosphate production, the modulation of reactive oxygen species, and the regulation of transcription factors involved in inflammation and cell survival [12–14]. Preclinical and clinical studies have demonstrated that PBM reduces edema, decreases the release of pro-inflammatory cytokines (e.g., TNF-α, IL-1β, and IL-6), and promotes both angiogenesis and tissue regeneration [14, 15]. Recent research has suggested that PBM can act synergistically with pharmacological and natural compounds, enhancing their therapeutic effects [16]. However, studies that explore the combined application of PBM and medicinal plants, such as *Desmodium heterophyllum*, are lacking, which justifies the relevance and innovation of the present work.

The aim of this study was to investigate the anti-inflammatory potential of the aqueous extract from *D. heterophyllum* in combination with photobiomodulation in the treatment of paw edema in mice submitted to a model of acute inflammation induced by carrageenan. The results of this study could contribute to the development of more effective, safer strategies for the treatment of acute inflammatory processes.

## Methods

### Plant collection

*Desmodium heterophyllum* was collected on the Mucuri campus of *Universidade Federal dos Vales do Jequitinhonha e Mucuri* (UFVJM) in the municipality of Teófilo Otoni, state of Minas Gerais, Brazil. Identification was performed by the DIAM Herbarium of the university and a voucher specimen was deposited under registration number DIAM10490. The material was registered and stored in the National System for the Management of Genetic Heritage and Associated Traditional Knowledge (SisGen) under registration number A22E930.

### Aqueous extraction

After drying and taxonomic identification, the plant material was crushed and ground. The aqueous extract was prepared by adding 400 mL of distilled water to 100 g of the powdered material; the same procedure was repeated for the leaves. Both preparations were stored in sealed containers for 24 h. The extracts were then heated to 55 °C for one hour and filtered at least twice using a vacuum pump. After preparation, the extracts were frozen at −80 °C for 24 h and freeze-dried for 24 h [17].

### Metabolic profiling of aqueous extract from D. heterophyllum using high-resolution mass spectrometry

The phytochemical extract was prepared at a concentration of 200 µg/mL and analyzed in a Q Exactive™ Orbitrap high-resolution mass spectrometer equipped with an electrospray ionization (ESI) source operating in positive ion mode (ESI⁺). Structural annotation of the major metabolites was carried out by comparing tandem mass spectrometry (MSⁿ) fragmentation data with both experimental data and in silico public spectral libraries. Molecular formula assignments were performed using the Xcalibur™ software, considering only formulas with mass errors less than 1 ppm. For determination of the molecular structure, the predominant ions detected in the mass spectra were subjected to fragmentation at collision energies of 20, 25, and 30 eV. Full-scans were performed over the m/z 100–1100 range. Structural identifications were based on characteristic MSⁿ fragmentation patterns and ion intensity profiles.

### Purification of heat shock protein 4 (HSP-4) and ecPis-2s

The synthesized peptides were purified using high-performance liquid chromatography (HPLC). The mobile phase consisted of a gradient mixture of a trifluoroacetic acid (TFA) solution in acetonitrile containing 0.08% v/v TFA and an aqueous solution containing 0.1% v/v TFA. Purification was performed in a Pro Star^®^ 315 chromatograph (Varian^®^), available in the UFVJM Chemistry Department. The system was equipped with a UV–Vis detector (Pro Star^®^ 335) and a Rheodyne^®^ injection valve. A 100-µL loop and Vydac^®^ C18 analytical column (250 × 4.6 mm) were employed to establish the purification conditions for the peptides. Chromatographic fractions corresponding to the compounds of interest were collected. Acetonitrile was removed from the collected fractions using a rotary evaporator (Fisatom^®^, model 801) in the UFVJM Chemistry Department. Subsequently, mass spectrometry analysis was performed. After initial analysis and confirmation of peptide synthesis by mass spectrometry, larger-scale purification was performed using a Waters mBondapak™ C18 semipreparative column (7.8 × 300 mm, 10 μm) with 1-mL injections of crude samples at a concentration of 1 mg·mL⁻¹ under optimized conditions for each peptide at a flow rate of 2 mL·min⁻¹. Detection was performed by UV–Vis spectroscopy at 215 nm. The isotopically labeled ecPis-2s peptide was purified in a Gilson chromatograph (model 215 Liquid Handler), with crude sample injections at a concentration of 10 mg·mL⁻¹. For all biophysical studies, the exact peptide concentration was determined spectroscopically [18, 19].

###  In vitro assay

J774A.1 and RAW 264.7 macrophages were cultured in supplemented RPMI-1640 medium (2 mM L-glutamine, 100 µg/mL streptomycin/penicillin, and 5% fetal bovine serum) at 37 °C in a 5% CO₂ atmosphere. Cytotoxicity was assessed using the 3-(4,5-dimethylthiazol-2-yl)−2,5-diphenyltetrazolium bromide assay (MTT test). Macrophages were plated (2 × 10⁵ cells/mL) in 96-well plates and incubated for 48 h (37 °C, 5% CO₂) in the presence or absence of the aqueous extract from *D. heterophyllum* (AEDh) at different concentrations (333, 100, 33.3, and 10 µg/mL). After 48 h, the supernatant was removed and 100 µL of supplemented Roswell Park Memorial Institute (RPMI) medium and 10 µL of MTT solution (5 mg/mL) were added to each well, followed by incubation for a further 4 h. The supernatant was then removed and the precipitate was diluted in 100 µL of dimethyl sulfoxide (DMSO). Absorbance was read in a plate spectrophotometer (EZ Read 2000, Biochrom, Holliston, MA, USA) at 570 nm. Viability (%) was calculated as (X1/X2)*100, in which X1 and X2 are respectively the mean optical density (OD) (560 nm) for treated and untreated cells. To determine nitric oxide (NO) production, the cells were cultured for 48 h in the presence or absence of AEDh (333, 100, 33.3, and 10 µg/mL) and stimulated with lipopolysaccharide (LPS) (1 µg/mL) and IFN-γ (0.9 ng/mL). After the incubation time had elapsed, the supernatants were collected for analysis of the NO concentration using the Griess method. One hundred µL of supernatant were transferred to 96-well plates and an equal volume of Griess reagent (1% sulfanilamide, 0.1% N-(1-naphthyl)-ethylene diamine hydrochloride, and 2.5% H₃PO₄) was added. The concentration of NO was determined by comparison with a standard solution of sodium nitrite read in the EZ Read 2000 at 540 nm.

### In vivo test

#### Animals and induction of paw edema

Female BALB/c mice were purchased and kept in ventilated cages. Filtered water and NUVILAB-CR1 feed were provided *ad libitum*. The experiment received approval from the institutional Animal Research Ethics Committee under protocol number 06–2023 R and all procedures were carried out in accordance with the principles of the Brazilian Code for the Use of Laboratory Animals.

The acute inflammation model followed a previously established protocol [20], with modifications. The concentration of the aqueous extract from *D. heterophyllum* (AEDh) was defined based on previous studies with species of the same family [21–24]. The mice were weighed and divided into ten groups (*n* = 5 animals/group). The paws measured with calipers (0 h). Sixty minutes before the edema was induced, the mice were treated or not with phosphate-buffered saline (PBS), AEDh (50, 125, or 250 mg/kg), or dexamethasone (0.5 mg/kg). Carrageenan (2.5%) was dissolved in phosphate-buffered saline (PBS) and injected (20 µL) into the plantar pad of the left hind paw. An equivalent volume (20 µL) of PBS was injected into the right paw and used as a control. One hour after injection, animals treated with PBS or AEDh (50, 125, or 250 mg/kg) were subjected to photobiomodulation (PBM) using a 780-nm infrared laser (Twin Flex Evolution Laser; MM Optics, São Carlos, SP, Brazil). The irradiation parameters were as follows: output power, 70 mW; irradiation time, 20 s; energy density, 35 J/cm²; beam spot area, 0.04 cm²; delivered energy, 1.4 J; and power density, 1.75 W/cm².

The right and left paws were measured 1, 2, 3, and 4 h after the injection of carrageenan. Edema was calculated using the formula: Change in paw thickness (mm) = [thickness (mm) (ΔPaw Thickness (mm)=T_Carrageenan_​−T_PBS​_) of paw injected with carrageenan - thickness (mm) of paw injected with PBS]. After the measurement at the 4th hour, the mice were euthanized under intraperitoneal anesthesia (ketamine 100 mg/kg and xylazine 10 mg/kg). The left paw was removed and frozen at −80 °C for subsequent cytokine analysis.

### Maceration of soft tissue of paw

The plantar pad was excised, weighed, and macerated with 1 mL of 0.4 M NaCl, 0.05% Tween 20 (Merck & Co. Inc., Whitehouse Station, USA), 0.5% bovine serum albumin, 0.1 M phenyl-methyl-sulfonyl fluoride, 0.1 M benzethonium chloride, 10 mM ethylene diaminetetraacetic acid, and 20 pM aprotinin. The macerates were homogenized and centrifuged at 10,000 rpm for 15 min at 4 °C. The supernatants were collected and frozen at −80 °C for the cytokine assay.

### Concentrations of IL-1β, TNF, IL-6, and IFN-γ

The supernatants of the macerates were assayed for cytokines (IL-1β, TNF, IL-6, and IFN-γ) using enzyme-linked immunosorbent assay (ELISA) (BD Biosciences Pharmingen, San Diego, CA, USA). For such, 96-well microplates were coated with 100 µL of primary antibodies diluted in specific buffer and incubated at 4 °C for 18 h. After washing and blocking, the samples and recombinant cytokines (100 µL/well) were incubated at 37 °C for 2 h. After washing, 100 µL of detection antibody was added, followed by incubation at 37 °C for 1 h. Another wash and incubation with streptavidin-peroxidase (50 µL, 1:200 in PBS) for 30 min (room temperature) were carried out. After washing, tetramethylbenzidine was added, followed by incubation for 20 min. The reaction was stopped with 2 N sulfuric acid. Readings were performed in the EZ Read 2000 at 450 nm and concentrations were determined using standard curves.

### Statistical analysis

Statistical analyses were carried out using GraphPad Prism version 5.0. The data were expressed as mean ± standard error of the mean of at least three independent experiments. Significant differences between groups were determined using ANOVA, the Kruskal-Wallis test, or two-way ANOVA, when appropriate. Differences were considered significant when *p* < 0.05.

## Results

### High-performance liquid chromatography

**Fig. 1:**
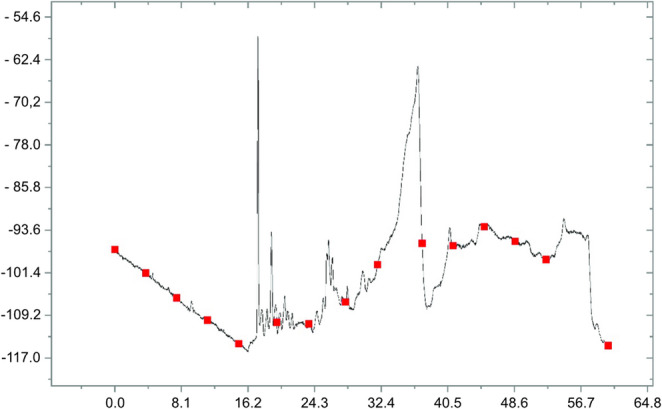
Chromatographic profile obtained after injection of 100 µL of a 1 mg·mL⁻¹ crude HSP-4 aqueous solution into a Vydac^®^ C18 column (250 mm × 4.6 mm)

The chromatographic profile obtained for the crude HSP-4 sample (1 mg/mL) demonstrated efficient separation in the C18 column, with a well-defined main peak, likely corresponding to the target peptide (Fig. 1). Elution was performed under optimized conditions using a TFA gradient in acetonitrile/water, indicating the effective removal of impurities. These results suggest that the purification method was suitable, enabling the collection of the desired fraction for subsequent mass spectrometry analysis and biophysical studies. The purity and identity of HSP-4 were confirmed, thus validating the protocol employed.

### High-resolution mass spectrometry (HRMS)-based metabolic profile of desmodium heterophyllum

**Fig. 2:**
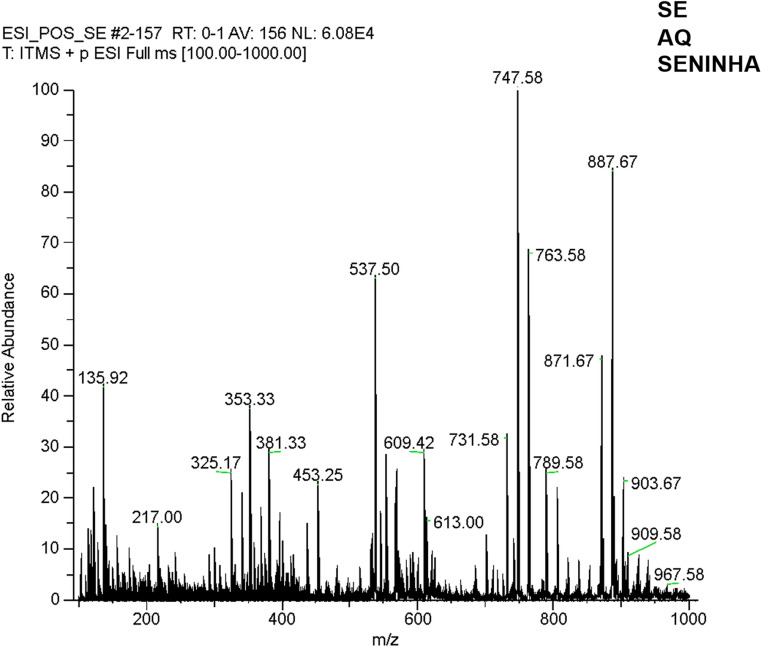
Mass spectrum of the aqueous extract acquired in an LTQ analyzer in positive ionization mode (ESI⁺)

The HRMS analysis of AEDh in positive electrospray ionization mode [ESI(+)] provided a detailed chemical fingerprint, with ions detected in the m/z range of 100–1100 (Fig. 2). Structural elucidation was performed by combining high-resolution mass accuracy (< 1 ppm) with MS/MS fragmentation patterns obtained at collision energies of 20, 25, and 30 eV. Most detected ions were concentrated between *m/z* 250 and 400, a region commonly associated with flavonoids and isoflavonoids, which are widely reported in the genus. Using the Xcalibur™ software, the molecular formula [C₁₅H₁₁O_5_]+, was assigned with high mass accuracy and its double bond equivalent (DBE) value and degree of oxygenation were consistent with oxygenated aromatic compounds of the flavonoid class. Additional formulas, such as C₁₅H₁₁O_4_] (m/z 255.06) and [C₁₅H₁₁O₆] (m/z 287.05), were identified as members of the same class, potentially representing liquiritigenin and quercetin/luteolin derivatives, respectively, which are markers frequently reported in the *Desmodium* genus. In the high-mass region (m/z > 800), the presence of glycosylated compounds was confirmed. The fragmentation of these ions often showed the neutral loss of 162 Da (hexose) or 146 Da (deoxyhexose), suggesting a profile of triterpenoid saponins. Additionally, low-mass ions (m/z < 200) and even-mass ions were detected, the latter indicating nitrogen-containing metabolites such as alkaloids or amino acids, which further contribute to the complex pharmacological activity of the extract.

### Cell viability and NO production

**Fig. 3:**
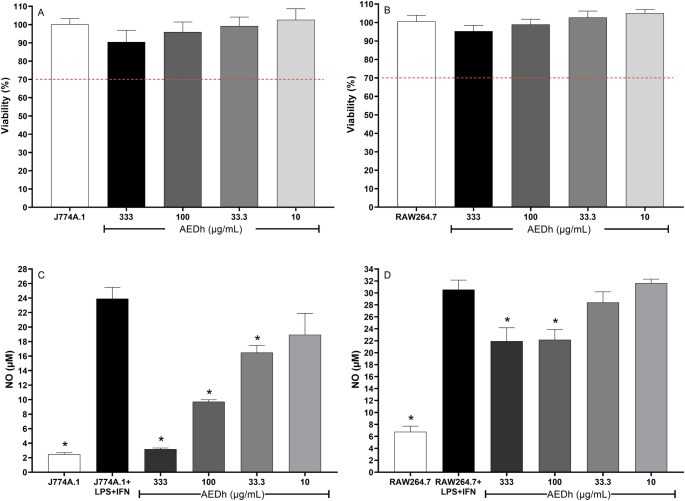
Effect of the aqueous extract of Desmodium heterophyllum (AEDh) on (**A**, **B**) cell viability and (**C**, **D**) nitric oxide (NO) production in J774A.1 (**A**, **C**) and RAW 264.7 (**B**, **D**) macrophages. Cells were stimulated or not with LPS+IFN-γ and treated for 48 hours with 10, 33.3, 100 or 333 µg/mL of AEDh. Each bar represents the arithmetic mean ± SEM of at least three independent experiments. *indicates *p* < 0.05 compared to the not-treated stimulated RAW 264.7 or J774A.1 control group, determined using ANOVA. Dashed line = cytotoxicity level according to ISO 10993-5:2009

The aqueous extract of the plant had excellent biocompatibility in the macrophage cell lines, with no cytotoxicity observed at any concentration tested (Fig. 3 A and B), without statistical differences being observed. AEDh at the highest concentrations reduced NO production in both cell lines. The reduction was even more significant in J774A.1, reaching 87.5% of reduction (3.18 ± 0.15 vs. 23.91 ± 1.56 µM) at the concentration of 333 µg/mL (Fig. 3 C and D).

### Analysis of variations in paw thickness

**Fig. 4:**
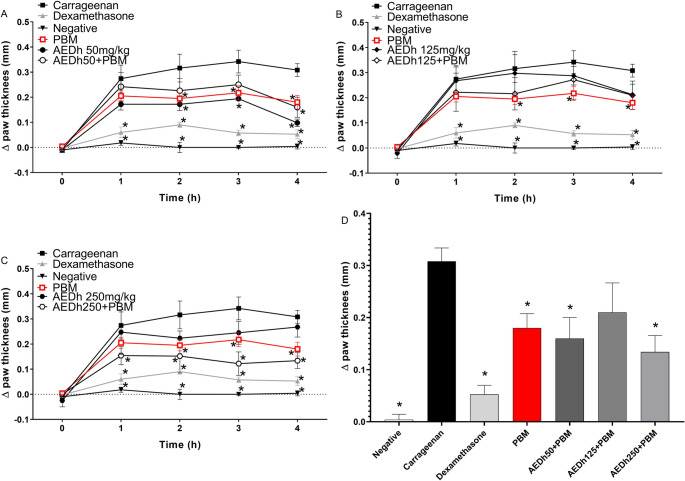
Effect of the aqueous extract of Desmodium heterophyllum (AEDh), photobiomodulation (PBM), and the combined AEDh+PBM treatment on paw thickness in the carrageenan-induced edema model. Variation in the thickness (mm) of the edema induced by carrageenan in the paws of female Balb/c mice (*n*=5/group) after 4 hours of evaluation. One hour before the edema induction, mice were treated with phosphated-buffered saline (PBS), 50, 125 or 250 mg/kg of the AEDh, or dexamethasone (5 mg/kg). One hour after the carrageenan injection, the animals were submitted to PBM. Each dot represents the arithmetic mean ± SEM. * indicates *p*<0.05 compared to induced and PBS-treated animals (Carrageenan), analyzed by two-way ANOVA with Dunnett’s correction. Negative = non-injected and not treated animals

Carrageenan-induced paw edema was evident from the first hour after injection (Fig. 4). When groups treated with different concentrations of AEDh in combination with photobiomodulation (PBM) were compared with PBM alone or AEDh alone, a similar reduction in paw thickness was observed (Fig. 4A–C). Notably, the combination of AEDh at 250 mg with PBM produced the greatest reduction in edema (Fig. 4 C), achieving levels comparable to those observed with dexamethasone treatment. Figure 4D shows the thickness of the paws in the fourth hour after induction, showing less edema with the combination of AEDh 250 and PBM compared to PBM alone (0.13 ± 0.03 vs. 0.18 ± 0.03 mm).

### Analysis of cytokine concentrations in macerated paws

**Fig. 5:**
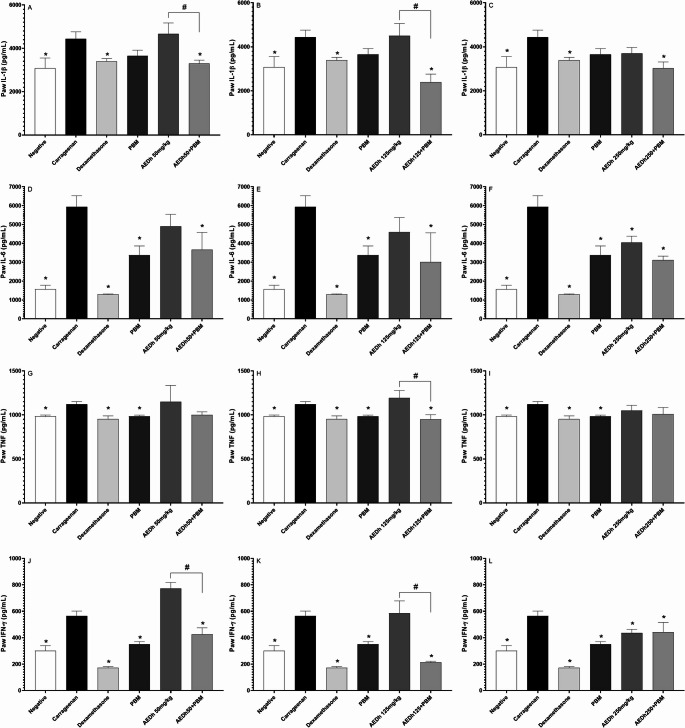
Modulation of pro-inflammatory cytokine levels (IL-1β, IL-6, TNF, and IFN-γ) in paw tissue following treatment with AEDh, PBM, and combined AEDh+PBM in the carrageenan-induced edema model

When IL-1β, IL-6, TNF, and IFN-γ were analyzed, the combination of PBM and AEDh reduced the production of the cytokines at all concentrations of the extract (Fig. 5) compared to the untreated group. The only exception was TNF. When analyzing IL-1β production, PBM alone failed to reduce cytokine production (3657.77 ± 258.11 vs 4432.03 ± 328.72 pg/mL), whereas the combination with the different concentrations of AEDh (3298.04 ± 158.31, 2395.02 ± 211.22, 3023.03 ± 289.44 vs. 4432.03 ± 328.72 pg/mL) was effective (Fig. 5 A, B, and C). AEDh alone was able to reduce the production of the cytokines IL-6 (4039.47 ± 213.71 vs. 5938.51 ± 586.02 pg/mL) and IFN-γ (441.71 ± 74.23 vs. 564.32 ± 37.61 pg/mL) at the concentration of 250 mg/kg (Fig. 5Fand L).

## Discussion

The present study demonstrated that the aqueous extract from *Desmodium heterophyllum* (AEDh) exerts anti-inflammatory activity both in vitro and in vivo, particularly when combined with photobiomodulation (PBM). The carrageenan-induced paw edema model, which is a well-established method for studying acute inflammatory processes, enabled an accurate assessment of the modulatory effects of the treatments tested and demonstrated the feasibility of combining herbal medicine with physical therapeutic resources.

AEDh significantly reduced NO production and the release of pro-inflammatory cytokines, demonstrating its immunomodulatory capacity. This effect is likely related to the suppression of the nuclear factor kappa B (NF-κB) signaling pathway, which regulates several pro-inflammatory mediators [25–27].

Mass spectrometry revealed the presence of phenolic compounds and flavonoids, which are metabolites widely recognized for their antioxidant and immunomodulatory properties. These findings are compatible with those described in previous studies that demonstrated the ability of flavonoids to inhibit NF-κB activation as well as inducible nitric oxide synthase (iNOS) and COX-2 expression [28–30].

In the in vivo experiments, treatment with AEDh significantly attenuated paw edema, with an early onset of action. Moreover, the combination with PBM enhanced this effect, suggesting a synergistic mechanism possibly related to the improved bioavailability of active compounds, modulation of local microcirculation, and regulation of intracellular signaling pathways [31–34]. Notably, the anti-inflammatory effect achieved with the highest concentration of the extract combined with PBM was comparable to that of dexamethasone, which is a standard corticosteroid, demonstrating the therapeutic potential of this integrative strategy, with the advantage of a lower risk of systemic side effects [31–34].

Another important aspect was the favorable safety profile of AEDh. Cytotoxicity assays revealed no reduction in macrophage viability at any of the concentrations tested, with viability consistently above 95%. This demonstrates the biocompatibility of the extract and its suitability for further preclinical development. Comparable safety profiles have been reported for other polyphenol-rich plant extracts, such as *Eugenia uniflora* and *Mangifera indica*, which also demonstrated minimal cytotoxicity and no genotoxicity [35–37]. Collectively, these results reinforce the potential for future clinical translation of the *D. heterophyllum* extract as a safe therapeutic agent.

Despite these promising results, some limitations must be acknowledged. The carrageenan model reflects acute inflammation and does not fully represent chronic or systemic inflammatory conditions. Moreover, no histopathological, antioxidant, or gene expression analyses were performed, which limits mechanistic insights. Future studies should address these gaps by incorporating chronic inflammation models, pharmacokinetic, histological and molecular analyses, and dose–response studies. Importantly, investigations into the long-term safety of the combination of PBM and AEDh as well as potential applications for conditions such as arthritis, dermatitis, and metabolic disorders are warranted.

In summary, this study provides novel evidence supporting the anti-inflammatory and immunomodulatory activity of the aqueous extract from *D. heterophyllum*, especially in combination with PBM, and demonstrates the potential of this combination as a safe, effective, integrative therapeutic approach for the management of inflammatory conditions.

## Conclusion

The results of this study indicate the anti-inflammatory potential of the aqueous extract from *Desmodium heterophyllum*, especially in combination with PBM, in a model of acute inflammation caused by carrageenan in mice. The combination significantly reduced edema, modulated pro-inflammatory cytokines, and exhibited low cytotoxicity. These findings suggest that the combination of herbal medicine and photobiomodulation may be an effective and safe therapeutic alternative comparable to dexamethasone.

## Data Availability

The data that support the findings of this study are available upon reasonable request.
